# Comparison of computed tomography‐guided percutaneous needle biopsy and endobronchial biopsy in the diagnosis of multifocal pulmonary lesions

**DOI:** 10.1002/jcla.22916

**Published:** 2019-05-10

**Authors:** Xiao‐Feng Li, Li‐Li Zheng, Yu He, Mao‐Shui Wang

**Affiliations:** ^1^ Department of Thoracic Surgery Shandong Provincial Chest Hospital, Shandong University Jinan China; ^2^ Central laboratory Liaocheng Peoples’ Hospital Liaocheng China; ^3^ Department of Clinical Laboratory First Affiliated Hospital of Guangxi Medical University Nanning China; ^4^ Department of Lab Medicine Shandong Provincial Chest Hospital, Shandong University Jinan China

**Keywords:** malignancy, sensitivity, specificity, tuberculosis

## Abstract

**Background:**

The retrospective study aimed to compare computed tomography (CT)‐guided percutaneous needle biopsy (PNB) and endobronchial biopsy (EB) in the diagnosis of multifocal pulmonary lesions with endobronchial involvement.

**Methods:**

Between November 2014 and June 2017, consecutive patients who had underwent both CT‐guided PNB and EB via bronchoscopy for diagnosis of pulmonary lesions were evaluated retrospectively. Tissue samples were submitted for pathological examination, acid‐fast bacilli, TB RT‐PCR, and mycobacterial culture. Sensitivities of the two methods alone or in combination were calculated and compared using Fisher's exact test.

**Results:**

Sixty‐seven patients (46 men and 21 women) were enrolled and could be diagnosed (32 malignant, 18 TB, and 17 benign). A final diagnosis of either malignant or TB diseases was made in 34 (68.0%) patients for CT‐guided PNBs, 19 (38.0%) patients for EBs, and 42 (84.0%) patients for the combination of both methods. Further statistical analysis showed significant difference in sensitivity between CT‐guided PNBs, or the combination of both methods, and EBs (all *P* < 0.05), and no difference between CT‐guided PNBs and the combination (*P* > 0.05). However, the combination of both methods appears to have the highest sensitivity in the detection of malignancies or TB diseases.

**Conclusion:**

Compared with EB, CT‐guided PNB has a high diagnostic yield for the detection of TB and malignancy in patients with multifocal pulmonary lesions with endobronchial involvement. When the two biopsies are combined, it appears to provide an incremental diagnostic value for the pulmonary lesions.

## 1 INTRODUCTION

Computed tomography (CT)‐guided percutaneous needle biopsy (PNB) is a minimally invasive procedure that is used to evaluate pulmonary lesions. CT‐guided PNB can establish a malignant diagnosis, establish a benign diagnosis, or obtain material for culture.^1^ Although there are several known complications including pneumothorax and hemorrhage, careful attention during biopsy planning, technique performance, and postprocedural care can help to prevent or minimize most potential complications.^2^ CT‐guided PNB has been considered an accurate approach for diagnosis. Geraghty PR et al reported the overall accuracy of CT‐guided PNB was 93.5%.^3^ However, central lesions adjacent to the bronchi remain one of the main relative contraindications to PNB.^1^

As an important tool in the diagnosis of bronchopulmonary diseases, bronchoscopy is preferred for central endobronchial or peribronchial lesions. Several studies supported that peripheral pulmonary lesions with endobronchial involvement are easily evaluated via bronchoscopy.^4^, ^5^ Moreover, endobronchial biopsy (EB) is a useful technique to diagnose infection when less invasive methods to diagnose the underlying etiology are not suitable; this may enhance the diagnostic utility of bronchoscopy in high tuberculosis (TB) burden countries, such as China and India.^6^

Imaging can aid in the selection of a technique to obtain tissue, including in the choice between bronchoscopic biopsy and PNB; however, in a proportion of patients who require tissue diagnosis, the optimal technique remains unclear for patients with multifocal pulmonary lesions with endobronchial involvement. In this study, we compared the diagnostic performance of these two methods to guide clinicians in the selection of the optimal technique.

## 2 METHODS

### 2.1 Subjects

At our institution, between November 2014 and June 2017, all consecutive patients who had undergone both CT‐guided PNBs and EBs via bronchoscopy for diagnosis of pulmonary lesions within 7 days were evaluated retrospectively. Demographic data were recorded, including age and sex. All data were collected using a medical record number unique to each member. Thus, all records used were de‐identified and anonymized.

### 2.2 CT‐guided PNB

The CT‐guided PNBs were performed using a semiautomated Tru‐Cut 18‐20 G needle (Precisa, Hospital Service, Aprilia, Italy) to obtain tissue specimens for histopathological diagnosis. At the request of the clinical departments, in cases of suspected TB, attempts were made to obtain samples for acid‐fast bacilli (AFB; auramine O stain), TB RT‐PCR (Daan), and mycobacterial culture (Lowenstein‐Jensen medium). Samples of the specimens obtained for cytology were immediately smeared on glass slides and fixed in 95% ethyl alcohol for preliminary evaluation by the cytotechnologist.

### 2.3 Endobronchial biopsy

Flexible bronchoscopy was performed using a fiberoptic bronchoscope (BF‐260, Olympus). EB (at least four specimens) was performed using biopsy forceps in the areas that appeared abnormal, if any, or from secondary carinal areas. Moreover, bronchial brushing was performed before EB. Biopsies were stained for mycobacteria. In addition to the stains, the specimens were also sent for TB RT‐PCR and mycobacterial culture.

**Figure 1:**
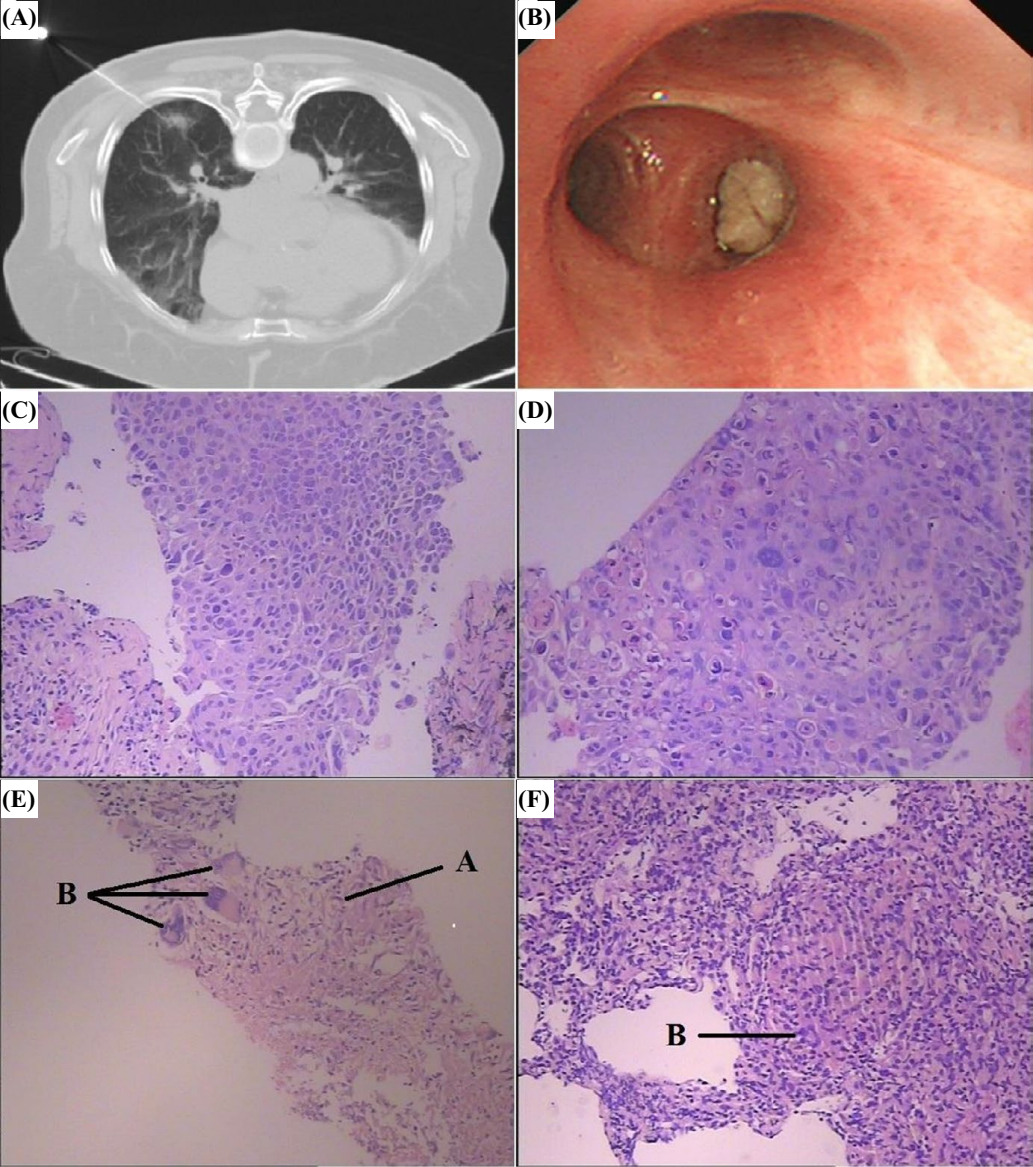
Computed tomography‐guided percutaneous needle biopsy of a lung lesion (A), and bronchoscopic finding revealing an endobronchial abnormality (B). A 65‐year‐old patient underwent computed tomography‐guided percutaneous needle biopsy (C), and endobronchial biopsy (D) was confirmed squamous cell carcinoma. A TB patient underwent computed tomography‐guided percutaneous needle biopsy (E), and endobronchial biopsy (F) was diagnosed with supportive histological evidence, such as epithelioid cells A and multinucleated giant cells B (hematoxylin and eosin stain, 200 × magnification)

The biopsy results were categorized as malignant, TB, and benign (Figure 1). The histopathological diagnosis of TB was based on the findings for epithelioid cells, multinucleated giant cells, or caseous necrosis.^7^ Benign diagnoses were categorized as specific if a benign neoplasm or specific infection (excluding TB) was diagnosed, and benign diagnoses were classified as nonspecific if the biopsy specimen showed nonspecific benign changes (eg, giant cells, leukocytes, histiocytes, inflammation, or fragments of fibrosis).

### 2.4 Statistical analysis

Continuous variables are expressed as the mean ± standard deviation. Categorical variables are presented as simple proportions. Sensitivities of the two methods alone or in combination were calculated separately. Fisher's exact test was used to evaluate the differences in sensitivity between pairs of categorical data. A *P* < 0.05 was considered significant. Analyses were performed using SPSS 16.0 (SPSS).

## 3 RESULTS

Of the 82 initially identified patients, 15 were excluded for the following reasons: inaccurate diagnosis (10 patients) and underwent examinations during different hospitalization periods (5 patients). Finally, sixty‐seven patients were enrolled in the study and could be diagnosed (32 malignant, 18 TB, and 17 benign). The mean age of the total patients’ cohort was 57.7 ± 12.4 years old. The patients consisted of 46 men and 21 women. Table 1 shows the characteristics of the patients and performances of CT‐guided PNB and EB in the diagnosis of pulmonary lesions.

**Table 1:** The performance of CT‐guided PNB and EB in the diagnosis of pulmonary lesions

	Malignant	Tuberculosis	Benign	Total
Number	32	18	17	67
Age (y)	61.4 ± 9.7	56.2 ± 13.7	52.2 ± 13.9	57.7 ± 12.4
Sex, male (%)	65.6% (21)	66.7% (12)	76.5% (13)	64.8% (46)
CT‐guided PNB				
Tissue	75.0% (24/32)	55.6% (10/18)	100.0% (17/17)	
Smear	45.2% (14/31)			
AFB	0.0% (0/18)	0.0% (0/13)	0.0% (0/9)	0.0% (0/40)
RT‐PCR	25.0% (1/4)	10.0% (1/10)	14.3% (1/7)	14.3% (3/21)
Mycobacterial Culture	0.0% (0/16)	13.3% (2/15)	0.0% (0/11)	4.8% (2/42)
EB				
Tissue	37.5% (12/32)	38.9% (7/18)	100.0% (17/17)	
Smear	21.9% (7/32)			
AFB	0.0% (0/24)	18.8% (3/16)	0.0% (0/15)	5.5% (3/55)
RT‐PCR	9.1% (1/11)	0.0% (0/12)	9.1% (1/11)	5.9% (2/34)
Mycobacterial Culture	0.0% (0/23)	8.3% (1/12)	0.0% (0/14)	2.0% (1/49)
CT‐guided PNB and EB				
Tissues	93.8% (30/32)	66.7% (12/18)		

Abbreviations: AFB, acid‐fast bacilli; CT, computed tomography; EB, endobronchial biopsy; PNB, Percutaneous needle biopsy; RT‐PCR, real time polymerase chain reaction.

Malignancy was diagnosed in 32 (47.8%) patients, and the mean age was 61.4 ± 9.7 years old; 21 of the patients (65.6%) were male. Seventeen patients (53.1%) had adenocarcinoma, six patients (18.8%) had squamous cell carcinoma, two patients (6.3%) had small cell lung cancer, and the remaining seven patients (21.9%) had other cancers. All patients underwent CT‐guided PNBs and EBs: CT‐guided PNBs yielded histological confirmation in 24 (75.0%, 95% CI: 57.9%, 86.8%) patients, and EBs yielded histological confirmation in 12 (37.5%, 95% CI: 22.9%, 54.8%) patients. Aspiration smears were performed for 31 patients and yielded a diagnostic sensitivity of 45.2% (95% CI: 29.2%, 62.2%). Bronchial brushings yielded a diagnosis of malignancy in 21.9% of the patients (7/32, 95% CI: 11.0%, 38.8%).

TB was diagnosed in 18 patients, and the mean age was 56.2 ± 13.7 years old; 12 of the patients (66.7%) were male. Six had extrapulmonary TB. All patients underwent CT‐guided PNBs and EBs: CT‐guided PNBs yielded histological confirmation in 10 (55.6%, 95% CI: 33.7%, 75.4%) patients, and EBs yielded histological confirmation in 7 (38.9%, 95% CI: 20.3%, 61.4%) patients. Cancer cells were negative in all aspiration smears and all bronchial brushing smears. Aspiration smear and bronchial brushing smear were examined for AFB in 13 and 16 patients, respectively; none of patients were positive in aspiration smears; and two patients (12.5%, 95% CI: 3.5%, 36.0%) were positive in brushing smears. TB RT‐PCR was performed in 10 CT‐guided PNBs and 12 EBs, and only one CT‐guided PNB yielded a positive result. Mycobacterial culture was performed in 15 CT‐guided PNBs and 12 EBs, and yielded two (13.3% 95% CI: 3.7%, 37.9%) and one (7.1%, 95% CI: 1.3%, 31.5%) positive results, respectively. In fact, the both methods made histological TB confirmation in 66.7% (12/18, 95% CI: 43.8%, 83.7%) of 18 patients, although combined with other tests including AFB smear, TB RT‐PCR, and mycobacterial culture, it appears to provide little incremental value in the diagnosis, since only one new case was identified.

In terms of detecting malignancy, the sensitivity of CT‐guided PNBs was superior to that of EBs (*P* < 0.05), and all patients with positive brushing cytology had positive needle biopsies. There was no difference in the sensitivity rates between EBs and bronchial brushings (*P* > 0.05). Compared with EBs, the sensitivity of CT‐guided PNBs was markedly higher (*P* < 0.01). Although the sensitivity of the combination of both methods (CT‐guided PNBs and EBs) appears to be higher than that of each one alone, there was no significant difference in sensitivity between the combination of both methods and CT‐guided PNBs (*P* = 0.082 > 0.05).

In terms of detecting TB, there was no difference in sensitivity rates between CT‐guided PNBs and EBs (*P* > 0.05); the combination was not superior to that of each one alone (*P* > 0.05).

For CT‐guided PNBs, a final diagnosis of either malignant or TB diseases was made in 34 (68.0%, 95% CI: 54.2%, 79.2%) patients; for EBs, a final diagnosis of either malignant or TB diseases was made in 19 (38.0%, 95% CI: 25.9%, 51.9%) patients; and for the combination of both methods, a total diagnosis of either malignant or TB diseases was made in 42 (42/50, 84.0%, 95% CI: 71.5%, 91.7%) patients. There was statistical difference in sensitivity between CT‐guided PNBs, or the combination, and EBs (all *P* < 0.05). Although there was no difference in the sensitivity between the combination of both methods and CT‐guided PNBs (*P* > 0.05), the combination appears to have the best sensitivity in the detection of malignancies or TB diseases.

In the EB group, there were 24 (24/67, 35.8%) patients reported with bleeding and all were controlled by blood aspiration and local instillation of cold saline lavage. In the CT‐guided PNB group, 12 (12/67, 17.9%) patients had an intrapulmonary hemorrhage and 10 (10/67, 14.9%) had pneumothorax, but only two patients (one hemorrhage and one pneumothorax) required treatment. No surgical interventions for these complications were needed, and no fatal events occurred.

## 4 DISCUSSION

Because of its advantages, which include being minimally invasive, easy and simple to handle, and low rate of complications, CT‐guided PNB has been widely used in the diagnosis of pulmonary lesions, especially for lung cancer, with high accuracy and safety.^8^ In the present study, we investigated the performance of CT‐guided PNB in the diagnosis of multifocal pulmonary lesions with endobronchial involvement, while comparing CT‐guided PNB with EB via bronchoscopy. In high TB burden countries, we found that the sensitivity of CT‐guided PNBs for a final diagnosis of either malignant or TB diseases was superior to that of EBs (68.0% vs 38.0%). If the two techniques were combined, the combination will greatly improve the diagnostic sensitivity for malignancies or TB diseases and lead to a more satisfactory detection result (42/50, 84.0%).

In contrast to bronchoscopic procedures, needle biopsy avoids contamination of the specimen. Additionally, with image guidance, the needle can be specifically directed toward the target lesion. However, when target lesions were adherent to the bronchi, it was considered a contraindication to needle biopsy. During bronchoscopy, a variety of sampling methods has been used. Usually, when visible airway abnormalities are present, endobronchial biopsy was preferred for a better assessment. Obtaining a tissue biopsy for pathological examination is key to ensuring a prompt and appropriate diagnosis and effective treatment for the pulmonary lesions. In a recent study that evaluated the diagnostic accuracy of CT‐guided PNB for pulmonary lesions, the diagnostic accuracy, sensitivity, and specificity of CT‐guided PNB were reported to be 92.9%, 95.3%, and 95.7%, respectively.^9^ In our study, CT‐guided PNB also showed good performance in the diagnosis of pulmonary lesions, and the detection rate could be greatly improved if CT‐guided PNB was combined with EB, reach as high as 90%. Although other tests, such as AFB smear, TB RT‐PCR, and mycobacterial culture, were performed with the biopsies, these tests appear to provide little incremental value in the diagnosis, since only one new case was identified. It is worth noting that in this study, two patients with malignancies were diagnosed with a comorbidity of pulmonary TB, and one of the patients was histopathologically confirmed by a sample from CT‐guided PNB, which originally missed the diagnoses of malignancy. However, considering the outcome, this patient was classified in the malignant group.

The complication rate and diagnostic performance are the two main factors to consider when choosing a diagnostic procedure. Until now, several complications have been reported in patients underwent PNB, including pneumothorax, hemorrhage, tumor seeding,^10^ and air embolism.^11^ Pneumothorax and hemorrhage are the most two common complications, with reported frequencies of 17%‐26.6% and 4%‐27%, respectively.^2^, ^12^, ^13^, ^14^, ^15^, ^16^ Many studies have revealed risk factors for developing complications, such as age, smoking habits, lesion size, adhesion to the pleura, and chronic obstructive pulmonary disease.^17^, ^18^, ^19^ In the study, 12 patients had intrapulmonary hemorrhage, and 10 had pneumothorax; only two patients (one hemorrhage and one pneumothorax) required treatment. In fact, most of the complications from CT‐guided lung biopsy were minor and acceptable, and the major complication rate was low for CT‐guided lung biopsy.^20^ For flexible bronchoscopy, the frequency of complications including hypoxemia, cardiac arrhythmias, bleeding, pneumothorax, fever, and infection was reported to be 5%−32%.^21^ In our study, there were 24 (35.8%) patients who underwent EB who reported bleeding, and all of the cases bleeding were controlled by blood aspiration and local instillations of cold saline lavage. Therefore, EB is considered to be a well‐tolerated procedure that can be performed safely on an outpatient.

There were several limitations to the current study, and these should be noted. First, retrospective design cannot exclude the possibility of bias, such as the experience of the operators and pathologists, characteristics of targeted nodules or endobronchial lesions, nonrandom sampling sequence, and easily assess results of the other test. Second, the relatively small sample size used may lead to an incorrect representation of the population, which also leads to the inability to identify specific risk factors of discordance between CT‐guided PNBs and EBs. Third, in the study, a relatively high proportion of TB patients were enrolled, which may reflect the fact that both biopsies have significant diagnostic value in high TB burden countries, such as China.^22^ Fourth, because of insufficient information, the tumor size was not measured by CT‐guided PNB and was not included in the analysis that estimated the association between tumor size and the diagnostic results. Fifth, it should be noted that some, more central lung lesions, are more amenable to EB. Therefore, patients with multifocal pulmonary lesions with peripheral and endobronchial lesions may benefit from the combination of the two methods.

## 5 CONCLUSIONS

In conclusion, pulmonary lesions can be of various etiologies. However, in high TB burden countries, TB and malignancy were more common and more severe. The present study indicates that compared with EB, CT‐guided PNB has a high diagnostic yield for the detection of TB or malignancies in the diagnosis of multifocal pulmonary lesions with endobronchial involvement. When the two methods are combined, it appears to provide an incremental diagnostic value for the pulmonary lesions. By a prompt diagnosis of the most patients, it permits initiation of specific therapies earlier. Finally, both the methods are minimally invasive surgical procedure, and the combination of both methods can diagnose TB and malignancy in patients with multifocal pulmonary lesions with endobronchial involvement effectively.

## CONFLICT OF INTEREST

The authors declare that they have no conflict of interest.
